# Assessing the effect of cleansing products on artificially polluted human hairs and skin through in vivo and in vitro models

**DOI:** 10.1111/srt.13220

**Published:** 2023-01-07

**Authors:** Anthony Galliano, Chengda Ye, Fengjie Su, Chad Wang, Rima Rakshit, Myriam Guerin, Frédéric Flament, Andrew Steel

**Affiliations:** ^1^ L'Oréal Research and Innovation Centre Charles Zviak Saint‐Ouen France; ^2^ L'Oréal Research and Innovation Shanghai China; ^3^ L'Oréal Research and Innovation Mumbai India

**Keywords:** antideposit, cleansing, hair, pollution, scalp, sebum

## Abstract

**Objective:**

Based on in vivo data, in vitro models and new methods are created to mimic the impact of aerial pollution onto the hair surface and assess the efficacy of different formulae prototypes.

**Material and methods:**

Two protocols are developed to mimic the pollution effect, in vitro, on purchased swatches, and in vivo, on scalps and forearms. First, with an artificial sebum mixed with Carbon Black particles, named “sebollution,” we evaluated, through an instrumental color measurement, the cleansing efficacy of some shampoo on scalp and hair. The second protocol allowed to assess the interaction between hair care product deposit (shampoo, conditioner, mask, and leave‐on) on hair and carbon black particles spread on fiber. The quantification of particle coverage allowed to evaluate the efficiency of a formula to limit the aerial pollution deposit on hair fiber.

**Results:**

To simplify and accelerate the evaluation of 42 shampoo formulae, an extrapolation of the scalp cleaning process was validated on forearm. The respective cleanabilities were calculated and covered a large range of efficacy, from 5%, for a basic bland shampoo generally used to reset swatches, to a strong deep cleansing efficacy of 100%. On hair swatches, cleanability efficiencies of five shampoo were also evaluated to eliminate the deposited of sebollution, in a range of 40%–80%. To quantify the efficacy of preventing the deposition of carbon particle on hair surface, the percentage of coverage of 45 different products was measured, from 2% to 16%. The performance depended of the product category (shampoo, conditioner, mask, and leave‐on), driven by the performance of the product deposit, and the capacity of this deposit to interact with aerial pollution.

**Conclusion:**

Three new protocols and evaluation methods are proposed to evaluate and quantify the performance of hair care product, to remove/clean, limit, and protect the hair fibers against the aerial pollution that could interact with hair, scalp and sebum. The validation of these approaches was done through the testing of a large panel of hair care product leading to a complete and sincere evaluation of cleansing and anti‐deposit efficacy. Combining the knowledge acquired on pollution impact on hair and the development of specific way of evaluation, this work reinforced the rationale of using and developing new cosmetic products that reduced the impact of pollution upon some hair properties.

## 1 INTRODUCTION

Aerial pollution (AP) that comprises gaseous elements with oxidative properties (NOx, SO_2_, O_3_..) and particulate matters (PMs) of various sizes (0.1, 1, 2.5, 10 μm and above) has become a serious concern in most developed countries and a source of anxiety.^1^ This led to an increasing amount of papers related to its impact(s) on different tissues (lungs, trachea) and its triggering effect on pre‐existing delicate conditions such as asthma, allergies etc.^2^, ^3^, ^4^, ^5^, ^6^ Other papers focused on the effect of AP on the skin physiology,^7^, ^8^, ^9^, ^10^, ^11^, ^12^, ^13^ observed in some cases by studying subjects living in two close Chinese cities differently polluted. This approach allowed to show that a more severe AP accentuates some facial signs, obvious markers of facial skin aging.^13^

The effect(s) of AP on the human hair received less attention, since viewed as a dead skin appendage, of minor physiological impact, that is, unthreatening the human health, albeit polluted hairs are most often perceived rough, dry, lack of shine/dull, as self‐declared in face to face interviews. In a previous paper,^14^ polluted human hairs were indeed shown presenting thousands of PM adhering to its surface, largely enhanced by the presence of sebum, making them duller and more difficult to manage, making such adherence a possible marker of AP severity. Interestingly, from an environmental aspect, an increasing number of works now focus on the counting of PM adhering to the leaves of different species of urban trees, as relevant indicator of AP.^15^

As daily dedicated to the development of hair care products, several issues remained to be investigated. How can the human hair be better protected from AP and how can it be de‐polluted with best performing hair care products were the main objectives of the present exploratory study. Based on in vivo data, some in vitro models were created to best mimic the impact of AP onto the hair surface. On an applied aspect, these in vitro models were used to assess the efficacy of prototypes of different formulae. The results of these investigations are the foci of the present paper.

## 2 MATERIAL AND METHODS

### 2.1 Polluting scalps and forearms in vivo

An artificial sebum was mixed with Carbon Black particles, (average size 2.07 μm ± 0.3 ‐ Hainuo Charcoal Co.Ltd. Shanghai, China ‐ CI 77266) and Iron Oxides (CI 77492) in a 9/1 proportion (w/w), respectively, referred here as “sebollution”.^16^

Note that 2 mg/cm^2^ of the same mixture was applied onto 12 delineated square areas (2 cm by 2 cm, 6 per forearm) of the forearms of 18 Chinese women (aged 18–40y) and onto small pre‐shaved areas (∼1cm^2^) at the vertex of their scalps. These procedures aimed at comparing the respective cleansing efficiencies of shampoo formulae on these two substrates, in an attempt to evaluate the relevance of forearms, as acceptable models in the pre‐selection of formulae. On forearms and scalps, the cleanability is computed using a Dermascore^R^ tool^17^, ^18^ in diffuse light illumination mode by measuring the number of dark pixels.

$$\text{Cleanability}\left(\%\right)=\frac{T_{\text{percentage}\text{of}\text{dark}\text{pixel}}^{0}-T_{\text{percentage}\text{of}\text{dark}\text{pixel}}^{1}}{T_{\text{percentage}\text{of}\text{dark}\text{pixel}}^{0}}$$

Note that 2 mg/cm^2^ of the sebollution is applied on each area, and pictures are taken at T0. The shampoo is then applied on each area, with a water ratio with product of 2:1 (w/w), mix and massage by fingerstall 30 circles for each area. The area is rinsed under shower for 30, and the excess of water is carefully wiped off using Kim‐Towel pads, without any transfer on the pad. A second picture is taken with Dermascore^R^ at T1. The analysis of picture by the image analysis of the Dermascore sotftware allows to determine the percentage of dark pixels. As for scalps, the shampooing procedure was performed under normal conditions of use: 10 g of the shampoo formula to be tested was applied onto previously wet hairs and scalp. Following a vigorous massage for 2 min, hairs and scalps were copiously rinsed under lukewarm tap water and dried using a hair‐drier before the recording of the colour of the tainted zone of the scalp.

### 2.2 “Polluting” hairs, in vitro

Note that 90 μl of the sebollution mixture was applied and spread manually, using gloves, all along the 18 cm of natural 100% white hair (1 g) supplied by HIP company (International Hair Importers & Products, White Plains, NY, U.S.A). This procedure aimed at mimicking the combination of sebum and adhesion of PM for assessing the cleaning potential of some shampoo formulae.^19^

**FIGURE 1:**
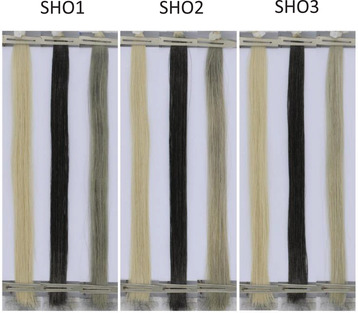
Examples of hairs swatches at three steps of their different phases (pre‐, after tainting, and postwash from left to right) for three different shampoo formulae SHO1‐2‐3, using pictures acquired by the Digi Eye BoxR system. The efficiencies of the different shampoo formulae (their cleanability) were calculated according to the following formula that was similarly used on scalps and forearms. The same formula was tested on six different hair swatches to ensure and evaluate the reproducibility of the results. L control corresponds to an untreated hair swatch.

Hair swatches were first wetted by lukewarm tap water and shampooed by 1 g of the formula under study for 1 min, copiously rinsed and dried off in an oven at 60°C for 1 h. The successive changes (pre, after tainting, and post wash) in the respective colours of hair swatches were assessed by a DigiEye Box system from VeriVide Company (VeriVide Limited Quartz Close, Warrens Business Park Enderby, Leicester, U.K). The latter instrument is a computer controlled digital imaging system that records color parameters (L*, a*, b* referential system) by capturing high quality repeatable images. Practically speaking, the L* value (Luminance, where L* = 0 corresponds to black) of a 7.2 cm by 0.6 cm area in the middle of hair swatches is recorded to evaluate the variable darkened status of hair swatches, pre‐ and postwash. This is illustrated by Figure 1, where three shampoo formulae were compared.

It clearly shows, at naked eye, that SH03 performs better than the 2 other shampoo formulae.

$$\mathrm{Cleanability}\left(\%\right)=\frac{L_{\mathrm{wash}}^{*}-L_{\mathrm{sebollution}}^{*}}{L_{\mathrm{control}}^{*}-L_{\mathrm{sebollution}}^{*}}\times 100$$

The average value of the standard deviation on the value of L*_control_ is about 1.

An in vitro approach to prevent or alleviate the deposition of PM on hairs.

**FIGURE 2:**
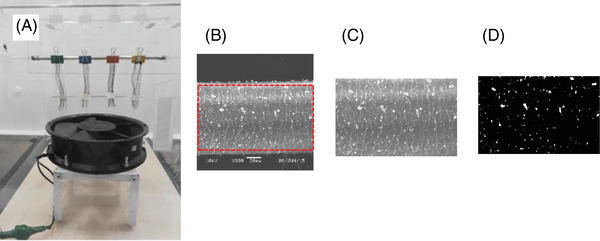
The successive steps used for artificially projecting CB particles onto hair swatches (A) and the processing of their SEM images corresponding to the SEM picture (B), magnification on the ROI defined (C), and the image analysis to evaluate particle coverage (D). CB, carbon black; ROI, region of interest; SEM, scanning electron microscopy.

A glove box (L = 80 cm, l = 50 cm, h = 60 cm, i.e., a volume of 0.24 m^3^) was used to dissipate and project carbon particles (2.5 μm) on vertically suspended natural hair swatches through a fan under constant flux, distant from the latter at 50 cm. Note that 0.1 g of hair was weighed and transformed into a flat swatch. These hair swatches were initially shampooed by various formulae under standard conditions (0.1 g of shampoo, massaged 2 min, copiously rinsed and dried‐off), one untreated swatch being used as control (absence of carbon black [CB] particles). The time of aerial projection was limited to 2 min. Hair swatches were carefully withdrawn from the glove box 15 min later, time for all CB particles to naturally fall on the base of the glove box by gravity. All along these testing procedures, for safety reasons, the dedicated operator wore a panoramic respiratory mask that covers the whole face, the glove box being located in a ventilated cabinet. Hair swatches were further observed by Scanning Electron Microscopy (JEOL JSM‐6060; Tokyo, Japan) aiming at counting the particles of 2.5 μm present onto the cuticular surface of 18 hair fibres per tested product. This was made possible by image analysis for eliminating the grey background of the hair surface, allowing an easier determination of the CB particles. Due to adhesive interactions between particles and product deposit on hair, some particles could be remained attached to hair. These were further expressed as a percentage of the covered hair surface. These successive steps and collected images are illustrated by Figure 2. The lower the coverage, the more efficient the formula in counteracting the deposition of CB particles.

## 3 RESULTS

“Polluting” scalps and forearms in vivo.

**FIGURE 3:**
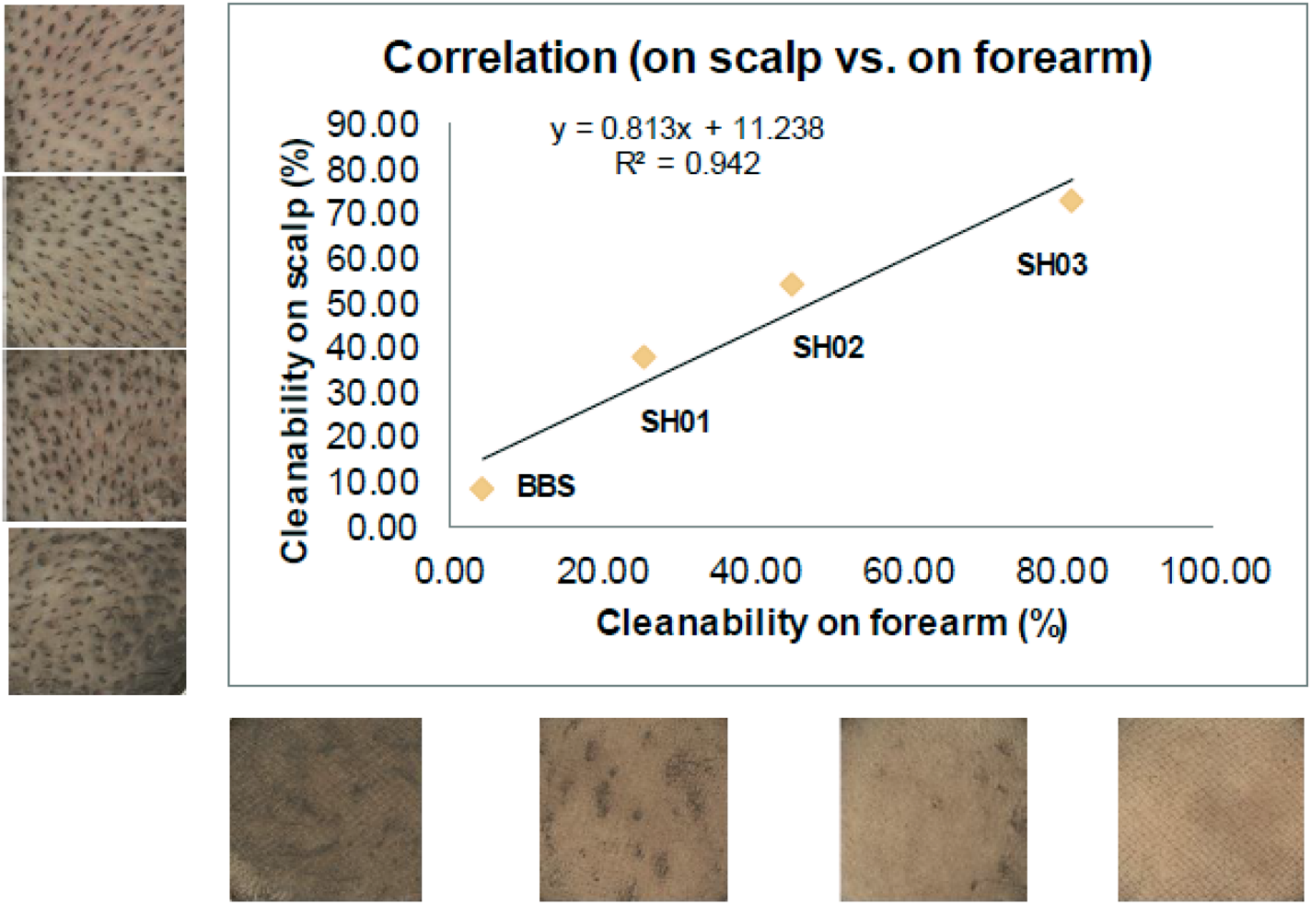
correlation between the two skin sites (scalp, forearm) of the different cleanabilities of four shampoo formulae

Among various shampoo formulae tested on forearms, four were arbitrarily chosen as offering the largest range in their cleanability values. These were then tested on scalp, posttainting a preshaved small scalp area (∼1cm^2^). They were applied as shampoos under the conditions previously mentioned. The correlation (*R* ^2^ = 0.942) between these two skin sites, illustrated by Figure 3, suggests that under such experimental conditions, the model of tainted forearm seems rather predictive of the scalp cleaning process, at least with the sebollution mixture used.

**FIGURE 4:**
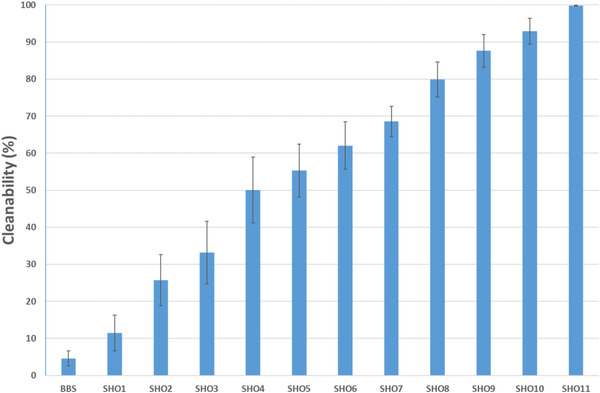
The various efficiencies (cleanabilities, mean ± SD) of 12 shampoo formulae (associated with pre‐shampooing or not) in eliminating the deposited association of sebum and CB on the forearm. CB, carbon black; SD, standard deviation.

Based on this extrapolation, 42 shampoo formulae were tested on the forearms, and their respective cleanabilities were calculated according to the aforementioned arithmetic formula. The 42 formulae comprised either preshampoos following their respective shampoos, or single shampoo formulae, being marketed or home‐made prototypes. Three hair swatches per formula were tested in blind under the same operating conditions, irrespective with their relative compositions. Figure 4 summarizes, by increasing order, the very different cleanabilities obtained on 12 representative formulae, ranging 5%–100%, arbitrarily coded as a function of their cleansing efficacies. This testing methodology appears reproducible as the standard deviation's (SD's) were of acceptable values, showing that these 12 formulae cover the widest range of cleanability in eliminating the sebollution mixture. Of note, the poor cleanability obtained for a basic bland shampoo (BBS) that was used to reset swatches prior use.

### 3.1 Polluting hair swatches in vitro

**FIGURE 5:**
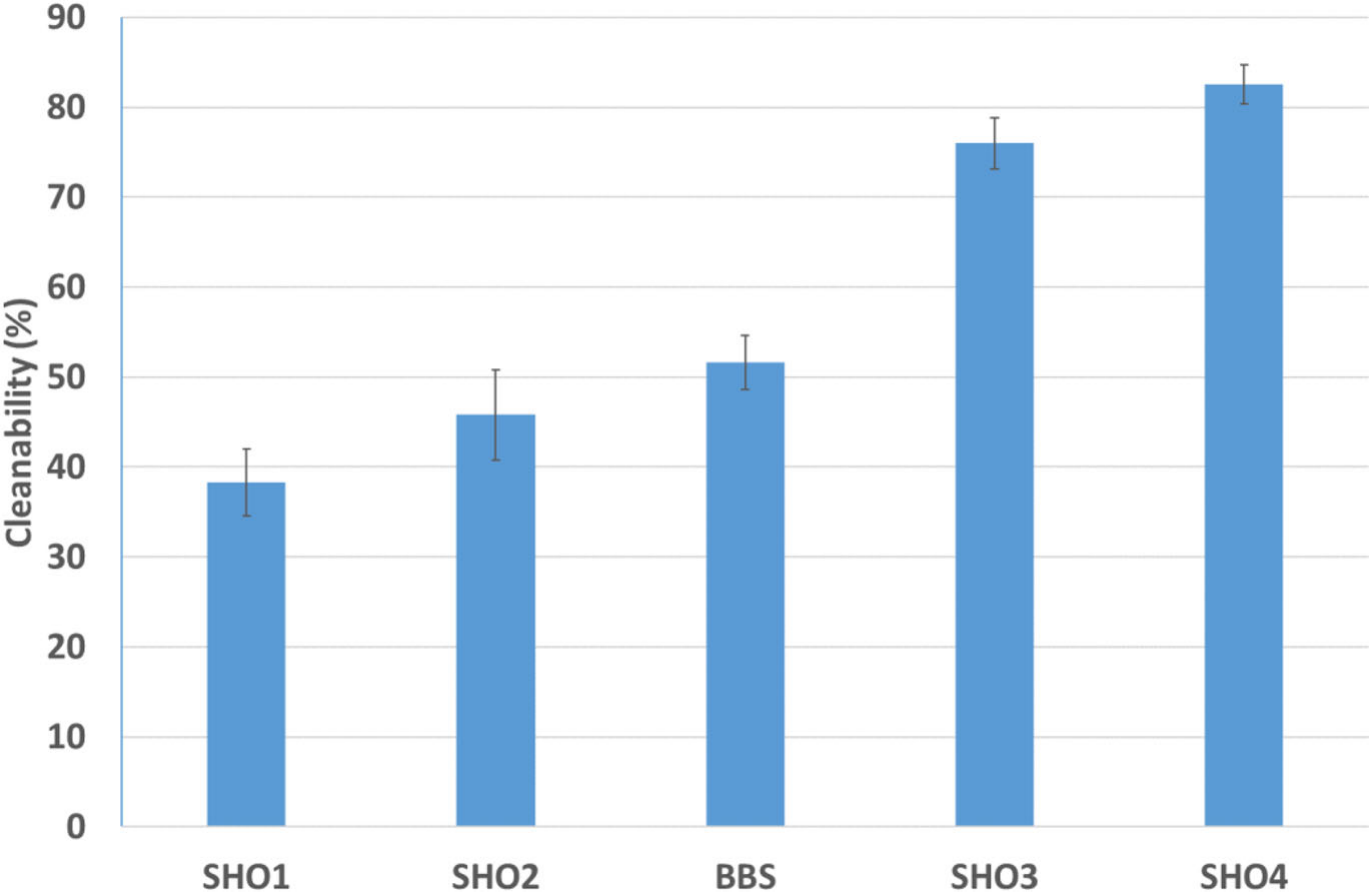
The various efficiencies (cleanabilities) of five shampoo formulae in eliminating the deposited association of sebum and CB on swatches

The measurement of cleansing on swatches was conducted with the DigiEye Box system measuring L* values. The Figure 5 showed some different technologies of shampoo tested to evaluate the cleanability of test shampoo against sebollution when applied on hair to mimic polluted hairs. The low performance (SH01) reaches a value close to 40%, whereas BBS shampoo reaches a medium performance (50% of cleanability on hair). The performance is different on hair and scalp but always positioned in the lower value of cleanability.

### 3.2 Alleviating/preventing the deposition of CB onto the hair surface

**FIGURE 6:**
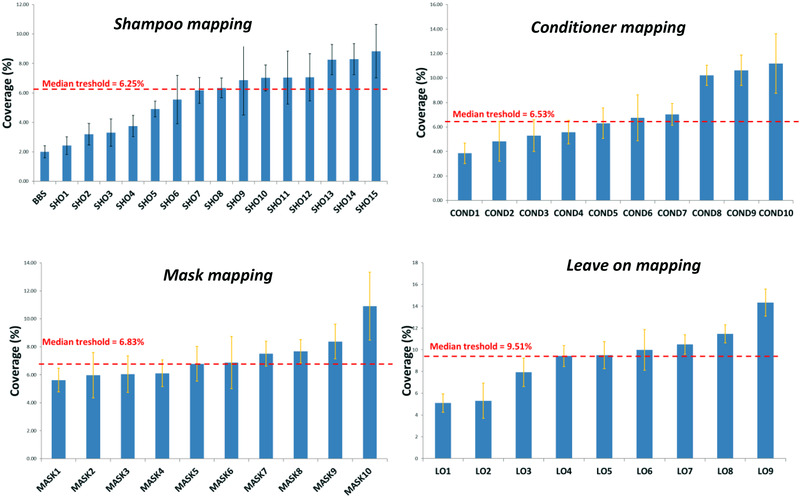
The various efficiencies of four categories of hair products in preventing or alleviating the deposition of aerial CB onto the hair surface. Red lines correspond to the median efficiency of each product category.

Forty‐five products were tested (shampoo, conditioners, masks and leave‐on) and mean efficiencies (as % of coverage) were calculated. These vary according to product category (6.2%–9.5%), allowing to differentiate products that perform better or less than the average. These are illustrated by Figure 6. Interestingly, a few shampoo formulae reached the lower values of CB coverage (2%–3%), as compared to leave‐on or mask products.

The result is a balance between the “cosmetic properties” (combing, smoothing, etc.), generally driven by the performance of the product deposit, and the capacity of this deposit to interact with AP. As reference, BBS shampoo reaches the lowest value, mainly linked to the fact that, free from conditioning agent, a deposit that interact with particles cannot be created.

## 4 DISCUSSION

A set of new and complementary methods to evaluate hair care product is proposed, aiming at removing, limiting and protecting the hair fibers against the combined assaults of aerial particles. Referred as sebollution, the present approach owns various positive aspects. At first, on a practical application, the promising correlation found between scalp and forearm, as model sites, clearly deserves further developments since carried out here on a very limited number of samples. Second, this methodology appears versatile as applicable to other objectives than the sole cleaning, that is, to alleviate/prevent the deposition of particles on many different products such as masks, leave on etc…

The results exposed in the present study seem fitting well with a recent study carried out in China^20^ that evaluated an intrinsic impact of pollution on the hair fiber. In this study, transmission electron microscopy cross sections of the tips of hairs were collected from subjects living in two Chinese cities (Baoding, more polluted, Dalian, less polluted). Images showed some fractures/gaps within the hair cortex. Their presences were found extremely variable, as quantified by image analysis. Their relative densities within the cross section were calculated and expressed as % of the total surface. It is showed that their median presence is about twice in Baoding than in Dalian and that some extreme cases (e.g., 0.5%–15%) may be found. Values below 2% were found highly frequent in hairs from Dalian, whereas almost absent in hairs from Baoding.

On a qualitative aspect, these gaps appeared mostly located within cellular remnants, close to melanosomes, suggesting that the oxidative processes (from the possible combination of pollution and ultraviolet [UV] exposure) led to a partial degradation of melanin grains at least at the tip regions under study. The major hair damage was found localized in cellular remnants, close to the melanosomes and at the nonkeratinized parts of the cuticle.

Hence, an additional or a synergetic effect between the presence of pollutant in or onto the hair, daily routines and UV exposure history could occur, most notably in the nonkeratinized parts. The higher total exposure to UV of tips could explain the higher observed hair damage, very likely amplified by an increased AP.

These results show the impact of pollution on hair sensitization and reinforce the rationale of the use of a cosmetic product that reduces the impact of pollution upon some hair properties. Some strategies are thus proposed to fight the “daily effects” and protect against “cumulative effect.” This antideposit method was shown covering a whole range of products. It allows, too, to evaluate both the antiadhesion effect and the one that counters the antideposition of pollution particles linked to the interaction between pollutant particles and the deposit of product onto the hair.

## CONFLICT OF INTEREST

All authors are the employees of the L'Oréal Group.

## Data Availability

None.
